# Altered Extracellular Vesicle MicroRNA Expression in Ischemic Stroke and Small Vessel Disease

**DOI:** 10.1007/s12975-018-0682-3

**Published:** 2019-01-07

**Authors:** Josie C. van Kralingen, Aisling McFall, Emily N. J. Ord, Thomas F. Coyle, Maria Bissett, John D. McClure, Christopher McCabe, I. Mhairi Macrae, Jesse Dawson, Lorraine M. Work

**Affiliations:** 1 Institute of Cardiovascular and Medical Sciences, College of Medical, Veterinary and Life Sciences, University of Glasgow, Glasgow, UK; 2 Institute of Neuroscience and Psychology, College of Medical, Veterinary and Life Sciences, University of Glasgow, Glasgow, UK; 3 BHF Glasgow Cardiovascular Research Centre, Institute of Cardiovascular and Medical Sciences, University of Glasgow, 126 University Place, Glasgow, G12 8TA UK

**Keywords:** miRNA, Ischemic stroke, TOAST subtype, Extracellular vesicle, Small vessel disease

## Abstract

Active transport of microRNAs (miRNA) in extracellular vesicles (EV) occurs in disease. Circulating EV-packaged miRNAs in the serum of stroke patients were compared to stroke mimics with matched cardio- and cerebrovascular risk factors, with corroboration of results in a pre-clinical model. An unbiased miRNA microarray was performed in stroke vs. stroke mimic patients (*n* = 39). Results were validated (*n* = 173 patients) by real-time quantitative polymerase chain reaction. miRNA expression was quantified in total serum/EV (*n* = 5–7) of naïve adult spontaneously hypertensive stroke-prone rats (SHRSP), their normotensive reference strain (Wistar Kyoto, WKY) and in circulating EV (*n* = 3), peri-infarct brain (*n* = 6), or EV derived from this region (*n* = 3) in SHRSP following transient middle cerebral artery occlusion (tMCAO). Circulating EV concentration did not differ between stroke and stroke mimic patients. The microarray identified many altered EV-packaged miRNAs: levels of miRNA-17-5p, -20b-5p and -93-5p (miRNA-17 family members) and miRNA-27b-3p were significantly (*p* ≤ 0.05) increased in stroke vs. stroke mimic patients. Patients with small vessel disease (SVD) consistently had the highest miRNA levels. Circulating EV concentration was unaltered between naïve SHRSP and WKY but levels of miRNA-17-5p and -93-5p were significantly increased in SHRSP. tMCAO in SHRSP did not further alter circulating EV miRNA-17 family member expression and nor did it change total miRNA-17 family levels in peri-infarct brain tissue or in EV isolated from this region at 24 h post-tMCAO. Changes in EV packaged miRNA expression was validated in patients with stroke, particularly those with SVD and corroborated pre-clinically. Together, altered circulating EV levels of miRNA-17 family members may reflect the chronic sequelae underlying cerebrovascular SVD rather than the acute ischemic stroke itself.

**Electronic supplementary material:**

The online version of this article (10.1007/s12975-018-0682-3) contains supplementary material, which is available to authorized users.

## Introduction

MicroRNAs (miRNAs) are a class of small, single-stranded regulatory RNAs, which post-transcriptionally regulate biological processes by modulating expression of target mRNAs. Over 60% of human protein-coding genes contain at least one conserved miRNA-binding site and the majority of protein coding genes are in-part regulated by miRNAs [1], the biological effects can be significant [2]. miRNA expression in blood is altered in people who have suffered ischemic stroke [3–7]. However, because comparisons were typically made with healthy controls, it is unclear whether these changes reflect stroke itself, risk factors for stroke or comorbid conditions.

Extracellular vesicles (EV) are lipid bilayer particles of endosomal origin, secreted as a result of the fusion of multivesicular bodies and the plasma membrane [8]. EV may be the primary mode of miRNA transport [9] and EV miRNA can mediate paracrine signalling [10]. The packaging and secretion of miRNAs in EV is active, better reflecting the pathobiology of disease than miRNAs released passively following cellular injury/necrosis. Changes in circulating EV expression of miR-9, miR-124 [11], miR-223 [12], miR-125, miR-422a [13], miR-21 and miR-30a [7] has been demonstrated in stroke patients but studies are limited by sample size, choice of control groups, lack of validation and a targeted/biased approach to screening.

In this series of studies, we profiled genome-wide circulating EV miRNA expression in people with suspected ischemic stroke, validated findings in a larger cohort and assessed changes in validated miRNAs in pre-clinical studies. We hypothesised that EV miRNA expression would differ in people with stroke compared to those with symptoms mimicking a stroke (stroke mimics, the non-stroke control group) and by stroke sub-type.

## Materials and Methods

### Clinical Study—Patient Recruitment

This study was approved by the Scotland A Research Ethics Committee (reference number 11/22/0077). All participants gave informed, written consent. All participants were reviewed within 24 h of first contact by a consultant stroke physician and discussed by a multi-disciplinary team. Stroke was diagnosed by consensus where presentation was consistent with stroke and supported by brain imaging. Stroke sub-type was assigned using criteria developed for the Trial of Org 10172 in Acute Stroke Treatment (TOAST) [14]. A non-stroke was diagnosed when presentation was not consistent with a vascular event and where an alternative firm diagnosis was made. These participants were used as our non-stroke, control group.

### Clinical Sample Collection

Peripheral blood samples were collected 48 h post symptom onset. To isolate serum, blood samples were allowed to clot for 20 min and then centrifuged at 3000*g* for 15 min at 4 °C before storage at − 80 °C.

### Preclinical Study—Experimental Animals

Male spontaneously hypertensive stroke prone (SHRSP) and their normotensive control, Wistar Kyoto (WKY) rats (270–310 g, 16–18 weeks old) were maintained ‘in-house’ by brother-sister mating. Research was conducted in accordance with the Animal Scientific Procedures Act 1986 incorporating European Directive 2010/63/EU and completed under PPL 60/4286. All studies were approved by the University of Glasgow’s Ethics Review Committee.

### Surgical Procedures

Rats were randomly allocated to sham or transient middle cerebral artery occlusion (tMCAO) procedure groups, anaesthetised (3% isoflurane in oxygen) and artificially ventilated. Four or 5 days prior to stroke (or sham) surgery, a cranial burr hole was drilled and durotomy performed [15]. SHRSP rats were subjected to tMCAO (*n* = 3–6) by advancing a silicone-coated monofilament (Doccol Corporation) via the internal carotid artery, blocking the origin of the MCA for 45 min before reperfusion, or sham procedure (*n* = 3–6) where the monofilament was advanced but immediately removed. Naïve age-matched WKY and SHRSP rats (*n* = 5–7) were included as controls.

### Sample Collection

Animals were terminally anaesthetised 24 h post-tMCAO or sham procedure or at an equivalent age (naïve). Blood samples were taken by cardiac puncture and serum isolated and stored as described for the clinical study. The infarct region and the cortical area immediately adjacent to this (peri-infarct) and equivalent regions on the contralateral hemisphere were dissected and immediately stored at − 80 °C.

### EV Isolation and Characterisation

EV were isolated from 200 μL of serum using total exosome isolation reagent (Thermo Fisher Scientific) according to manufacturer’s instructions. Brain-derived EV were isolated according to protocol [16] with the isolated pellet resuspended in phosphate-buffered saline. EV were visualised and their concentration quantified using nanoparticle tracking analysis (NTA) on a NanoSight LM10 (Malvern) and by transmission electron microscope (TEM) following resuspension in 2% paraformaldehyde. EV were subsequently fixed onto Formvar-carbon-coated electron microscope grids according to protocol [17].

### RNA Extraction

RNA was extracted from serum-derived EV (human or rat), 50 mg brain tissue, or from brain-derived EV using the miRNeasy Mini Kit (QIAGEN) according to manufacturer’s instructions with a DNase step performed for tissue extractions. The RNA concentration and quality was determined using a NanoDrop™ (ND-1000 spectrophotometer; Thermo Fisher Scientific).

### miRNA OpenArray™ Experimental Design and Statistical Analysis

cDNA was synthesised using the TaqMan™ MicroRNA Reverse Transcription Kit (Applied Biosystems) according to manufacturer’s instructions (*n* = 39 patients). The optimised low-sample input protocol was used [18]. To prevent systematic bias, samples were randomised across 13 TaqMan™ OpenArray™ Human MicroRNA Panels using R (https://www.r-project.org/). Then, 10 ng RNA was loaded into each reaction and spiked with 2.5 ng of an exogenous spike-in miRNA control (*Arabidopsis thaliana*: *ath-miR-159a*). cDNA was stored at − 20 °C until pre-amplification was performed. cDNA was pre-amplified using TaqMan™ PreAmp Master Mix and Megaplex™ PreAmp Primer Mix (pool A or B) according to manufacturer’s instructions. Pre-amplified cDNA was subsequently diluted 1:20 using 0.1X Tris-EDTA buffer (1 mM Tris and 0.1 mM EDTA, pH 8.0) and stored at − 20 °C until the OpenArray™ experiment was performed. Pre-amplified cDNA was loaded onto TaqMan™ OpenArray™ Human MicroRNA Panels according to manufacturer’s instructions using a QuantStudio™ 12 K Flex AccuFill™ System. Loaded microRNA panels were then placed in an OpenArray™ real-time PCR instrument and panels underwent thermal cycling at 50 °C for 2 min, 95 °C for 10 min and 95 °C for 15 s and 60 °C for 1 min, repeated for another 39 cycles.

In the OpenArray™ study, and validation experiments, the experimenter was blinded to the patient subtype and unblinded at data analysis. Data was initially analysed using DataAssist™ software. While all samples were screened for 754 human miRNA sequences, individual miRNAs were only taken forward for further analysis if detected in ≥ 30% of patients within any one stroke subtype (93 miRNAs met this criterion). Data were normalised to the exogenous spike-in control (*ath-miR-159a*) and ∆Ct values calculated. Comparisons were made first between ∆Ct values of all ischemic stroke patients vs. non-stroke controls and then between individual stroke subtypes and non-stroke controls. A two-tailed Student’s unpaired *t* test was used. Statistical significance was taken at *p* < 0.05. Thirteen miRNAs whose expression was significantly altered, as assessed by *t* test, were taken forward for validation.

### Bioinformatic Analyses

miRWalk 2.0 [19] was used to generate predicted gene targets for each miRNA in the miR-17 family. Targets predicted by seven or more of the databases used by miRWalk 2.0 software were used. Gene targets were subsequently uploaded to DAVID Bioinformatics Resources 6.8 [20] and the biological processes tool used to group together related gene targets.

### Quantitative Real-Time PCR

Samples from a total of 173 participants (including those screened in the OpenArray™ study) were included in the clinical validation study. Preclinically, expression of miRNA-17-5p, -20b-5p and -93-5p in naïve WKY and SHRSP (*n* = 5–7/group) and in SHRSP subjected to tMCAO (45 min; *n* = 3–6) or sham procedure (*n* = 3–6) was determined. cDNA was synthesised by RT-PCR from the total RNA of isolated EV using the TaqMan™ MicroRNA Reverse Transcription Kit according to manufacturer’s instructions. Each reaction contained 5 ng *Caenorhabditis elegans miR-39* (*cel-miR-39*) (exogenous spike-in miRNA control) and 5 ng of sample RNA. cDNA was stored at − 20 °C until used. When performing qRT-PCR on miRNA isolated from human EV, pre-amplification was performed using TaqMan™ PreAmp Master Mix according to manufacturer’s instructions. To profile miRNA expression, qRT-PCR was performed using TaqMan™ miRNA assays according to manufacturer’s instructions. Duplicate reactions were run for each sample on a 384-well plate.

### Statistical Analysis

When comparing qualitative demographic variables between groups, Fisher’s exact test was used. A Student’s *t* test, Mann-Whitney test or Kruskall-Wallis was used to compare quantitative demographic variables across groups. Relative quantification (RQ; fold change) was calculated following normalisation to exogenous spike-in control miRNA (*cel-miR-39*). Data are presented as RQ ± RQmax/RQmin relative to the expression in the control non-stroke (clinical studies) or WKY/sham (preclinical studies) group, whose expression was 1. Expression was first compared in all ischemic stroke patients vs. non-stroke controls and then between individual stroke subtypes and non-stroke controls. An unpaired two-sample Student’s *t* test or one-way ANOVA with post hoc Dunnett’s test was used for the clinical studies. Preclinical groups were compared using unpaired two-sampled Student’s *t* test (with the addition of a Bonferroni’s multiple comparisons test where appropriate). The data were analysed on the ΔCt scale. *p* < 0.05 was deemed statistically significant.

## Results

### Clinical Study

A total of 173 participants were recruited between 31 January 2012 and 20 April 2014. The OpenArray™ included 39 participants: (non-stroke (*n* = 10), large artery (*n* = 9), cardioembolic (*n* = 10) and small vessel disease (SVD; *n* = 10) patients). The validation study included all 173 patients: non-stroke (*n* = 34) and stroke (*n* = 139) patients. The stroke patients were further split according to TOAST with large artery (*n* = 22), cardioembolic (*n* = 40), SVD (*n* = 37) and unclassified (*n* = 40). Baseline characteristics are shown in Table 1 (OpenArray™ population) and Table 2 (validation cohort). There were no differences in baseline characteristics between stroke and non-stroke patients in the OpenArray™ population (Table 1) or in the validation cohort, although measures of stroke severity were higher in stroke patients as expected (Table 2). Baseline characteristics by stroke subtype are shown in Online Resource 1.

**Table 1:** OpenArray™ study patient characteristics

		Non-Stroke		Stroke		*p* (Fisher’s test)
		*n*	%	*n*	%	
Age and gender	*n*	10		29		*NA*
	Male, % (*n*)	7	70.0	22	75.9	0.696
	Median age [IQR]	70.0 [62.8–84.0]	–	73.0 [60.5–79.5]	–	0.544^✞^
Risk factors for ischaemic stroke	Hypertension, % (*n*)	7	70.0	15	51.7	0.464
	Atrial fibrillation, % (*n*)	3	30.0	5	17.2	0.399
	Diabetes (type 1 or 2), % (*n*)	3	30.0	7	24.1	0.696
	Hyperlipidaemia, % (*n*)	3	30.0	9	31.0	1.000
	Peripheral vascular disease, % (*n*)	0	0.0	0	0.0	1.000
	Smoker, % (*n*)	3	30.0	9	31.0	1.000
	Ex smoker, % (*n*)	3	30.0	6	20.7	0.669
	Previous stroke, % (*n*)	2	20.0	4	13.8	0.636
	Family history, % (*n*)	1	10.0	4	13.8	1.000
Medication	Recombinant tissue plasminogen activator, % (*n*)	–	–	10	34.5	NA
	ACE inhibitor, % (*n*)	4	40.0	5	17.2	0.197
	Alpha blocker, % (*n*)	1	10.0	0	0.0	0.256
	Anticoagulant, % (*n*)	1	10.0	1	3.4	0.452
	Antiplatelet, % (*n*)	6	60.0	8	27.6	0.124
	ARB, % (*n*)	0	0.0	3	10.3	0.556
	Beta blocker, % (*n*)	4	40.0	9	31.0	0.704
	Blood pressure treatment, % (*n*)	7	70.0	15	51.7	0.464
	CCB, % (*n*)	4	40.0	3	10.3	0.057
	Loop diuretic, % (*n*)	3	30.0	4	13.8	0.344
	Oral hypoglycaemic drugs, % (*n*)	2	20.0	2	6.9	0.267
	Spironolactone, % (*n*)	1	10.0	1	3.4	0.452
	Statin, % (*n*)	6	60.0	13	44.8	0.480
	Thiazide, % (*n*)	2	20.0	2	6.9	0.267
Stroke status	Median baseline NIHSS [IQR]	1 [0–5.5]	–	4 [2.5–11]	–	0.054^♦^

Demographical data for the full cohort of patients used in the OpenArray™ study. For dichotomous variables, a Fisher’s exact test was used to assess differences. For continuous variables, an unpaired two-tailed Student’s *t* test (✞) or Mann Whitney *U* test (^♦^) was used

*ACE* angiotensin-converting enzyme, *ARB* angiotensin receptor blocker, *CCB* calcium channel blocker, *NIHSS* National Institutes of Health Stroke Scale

**Table 2:** Validation study patient characteristics

		Non-Stroke		Stroke		*p* (Fisher’s Test)
		*n*	%	*n*	%	
Age and gender	*N*	34	–	139	–	NA
	Gender: male	19	55.9	90	64.7	0.428
	Median age [IQR]	63.5 [53.8–68.0]	–	68.0 [57.0–76.0]	–	0.055^✞^
Risk factors for ischaemic stroke	Hypertension, % (*n*)	13	38.2	57	41.0	0.847
	Atrial fibrillation, % (*n*)	3	8.8	27	19.4	0.206
	Diabetes (types 1 or 2), % (*n*)	7	20.6	24	17.3	0.625
	Hyperlipidaemia, % (*n*)	6	17.6	36	25.9	0.378
	Peripheral vascular disease, % (*n*)	2	5.9	1	0.7	0.099
	Smoker, % (*n*)	9	26.5	45	32.4	0.544
	Ex-smoker, % (*n*)	11	32.4	23	16.5	0.053
	Previous stroke, % (*n*)	5	14.7	19	13.7	1.000
	Family history, % (*n*)	4	11.8	22	15.8	0.789
Medication	Recombinant tissue plasminogen activator, % (*n*)	–	–	39	28.1	NA
	ACE inhibitor, % (*n*)	8	23.5	32	23.0	1.000
	Alpha blocker, % (*n*)	0	0.0	1	0.7	1.000
	Anticoagulant, % (*n*)	2	5.9	8	5.8	1.000
	Antiplatelet, % (*n*)	14	41.2	51	36.7	0.694
	ARB, % (*n*)	1	2.9	9	6.5	0.689
	Beta blocker, % (*n*)	10	29.4	35	25.2	0.664
	Blood pressure treatment, % (*n*)	14	41.2	68	48.9	0.449
	CCB, % (*n*)	5	14.7	25	18.0	0.803
	Loop diuretic, % (*n*)	1	2.9	15	10.8	0.202
	Oral hypoglycaemic drugs, % (*n*)	2	5.9	10	7.2	1.000
	Spironolactone, % (*n*)	1	2.9	2	1.4	0.484
	Statin, % (*n*)	16	47.1	60	43.2	0.704
	Thiazide, % (*n*)	3	8.8	15	10.8	1.000
Stroke status	Median baseline NIHSS [IQR]	1 [0–3]	–	4 [2–7]	–	< 0.0001^♦^

Demographical data for the full cohort of patients used in the validation study. For dichotomous variables, a Fisher’s exact test was used to assess differences. For continuous variables, an unpaired two-tailed Student’s *t* test (✞) or Mann Whitney *U* test (♦) was used

*ACE* angiotensin-converting enzyme, *ARB* angiotensin receptor blocker, *CCB* calcium channel blocker, *NIHSS* National Institutes of Health Stroke Scale

**Fig. 1:**
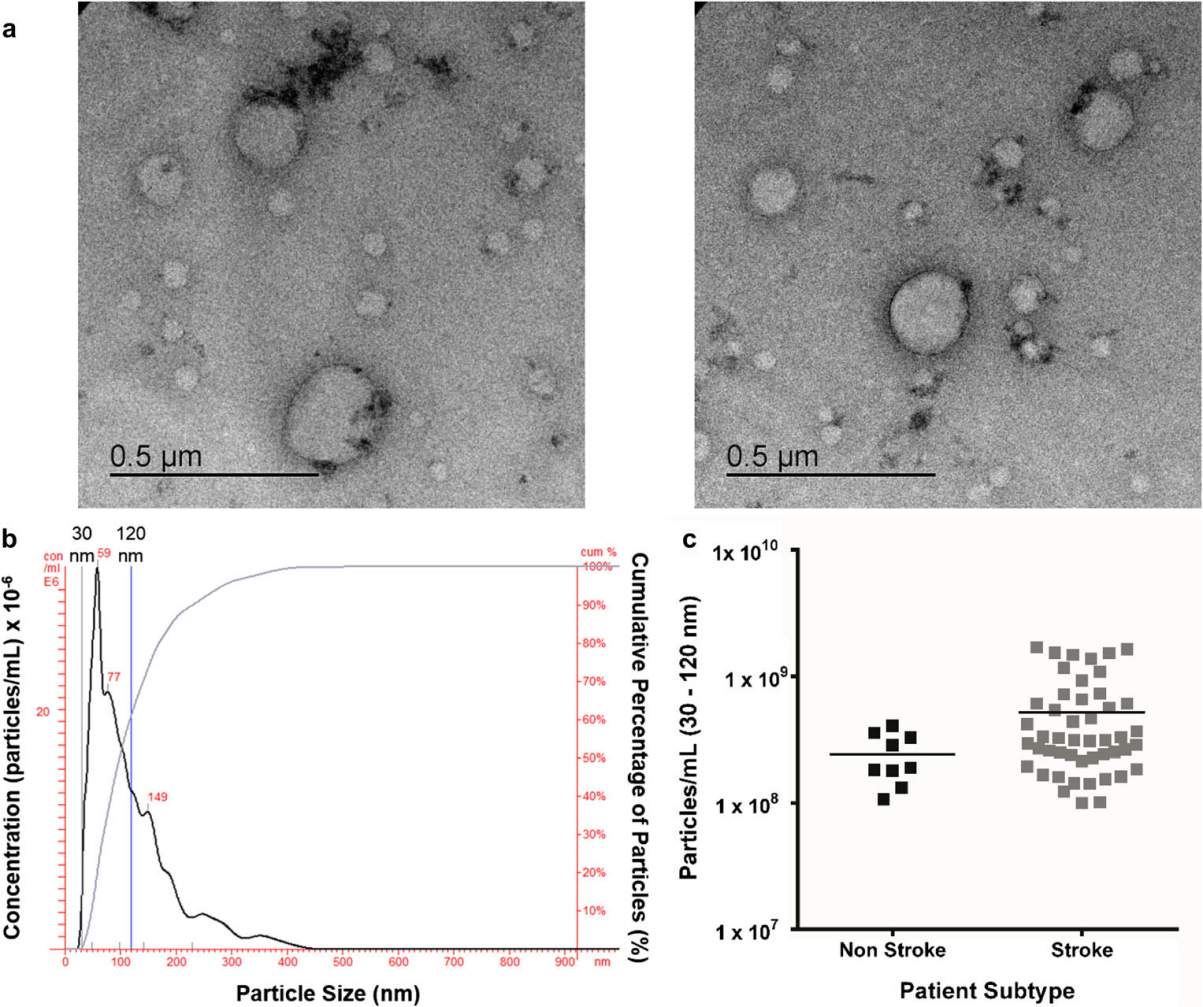
Extracellular vesicle visualisation and quantification. **a** TEM observation of whole-mounted EV purified from human serum by precipitation. **b** NanoSight trace for representative sample is shown. Black trace indicates EV concentration with increasing particle size, and blue trace shows cumulative percentage of EV with increasing particle size. **c** Concentration of EV (30–120 nm) in stroke (*n* = 48) and non-stroke (*n* = 9) patients. Horizontal bar represents mean. Unpaired two-tailed Student’s *t* test assessed statistical significance

EV isolated from serum of non-stroke and ischemic stroke patients at 48 h post-stroke were visualised by TEM (Fig. 1a) and size range confirmed by NTA (Fig. 1b). The mean number of serum-isolated EV of non-stroke and stroke participants did not differ (stroke 5.2 ± 0.7 × 10^8^ particles/mL vs. non-stroke 2.4 ± 0.4 × 10^8^ particles/mL (*t*_55_ = 1.8; *p* = 0.079) (Fig. 1c).

### OpenArray Detects Altered EV miRNA Expression in Ischemic Stroke Patients

**Fig. 2:**
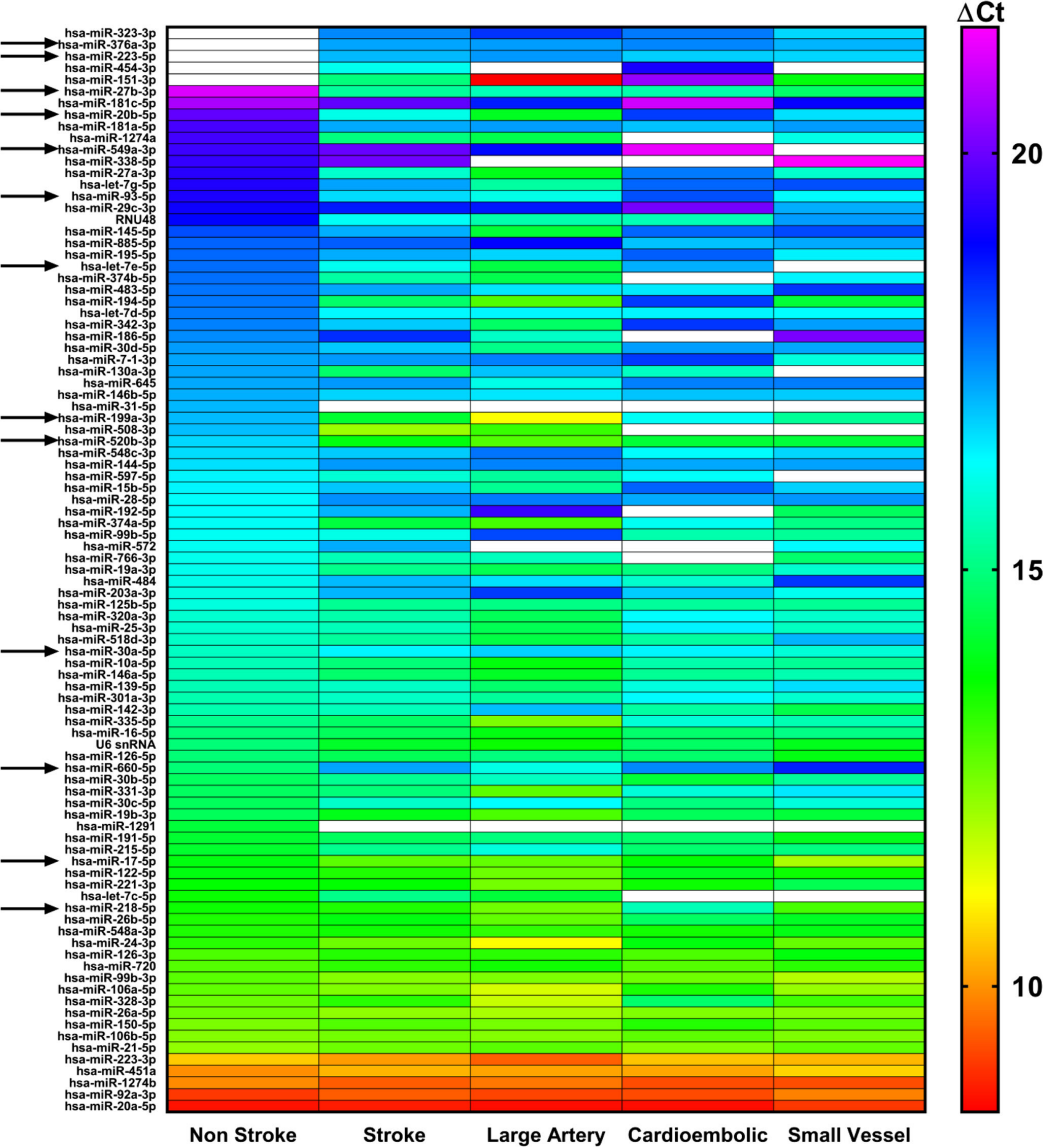
EV Expression of miRNAs Altered in Stroke Patients (OpenArray™). miRNA expression in EV from serum of control non-stroke patients (*n* = 10), all stroke patients (*n* = 29) and stroke patients separated by TOAST subtype: large artery (*n* = 9), cardioembolic (*n* = 10) and SVD (*n* = 10) stroke patients. Changes in EV miRNA expression were assessed by OpenArray™, data shown are average ΔCt values following normalisation to spike housekeeper, *ath-miR-159a*. Arrows indicate miRNAs taken forward for further investigation

There was significant upregulation of miRNA-27b-3p, -93-5p and -520b-3p and downregulation of miRNA-660-5p in EV from all ischemic stroke patients vs. non-stroke controls (Fig. 2). Other miRNAs were altered (up- or downregulated) in EV from patients with specific stroke subtypes (Fig. 2); miRNA-20b-5p, -30a-5p and -93-5p (large artery); miRNA-218-5p, -520b-3p and -660-5p (cardioembolic) and miRNA-17-5p, -93-5p, -199a-3p and -660-5p (SVD). In other cases, miRNA expression appeared to be either ‘switched on’ or ‘switched off’ in EV from ischemic stroke patients vs. non-stroke controls (Fig. 2). The 13 miRNAs whose expression appeared altered and that were taken forward for validation in a larger patient cohort were miRNA-17-5p, -20b-5p, -27b-3p, -30a-5p, -93-5p, -199a-3p, -218-5p, -223-5p, -376a-3p, -520b-3p, -549a-3p, -660-5p and -let-7e-5p.

### qRT-PCR Validates OpenArray Results

**Fig. 3:**
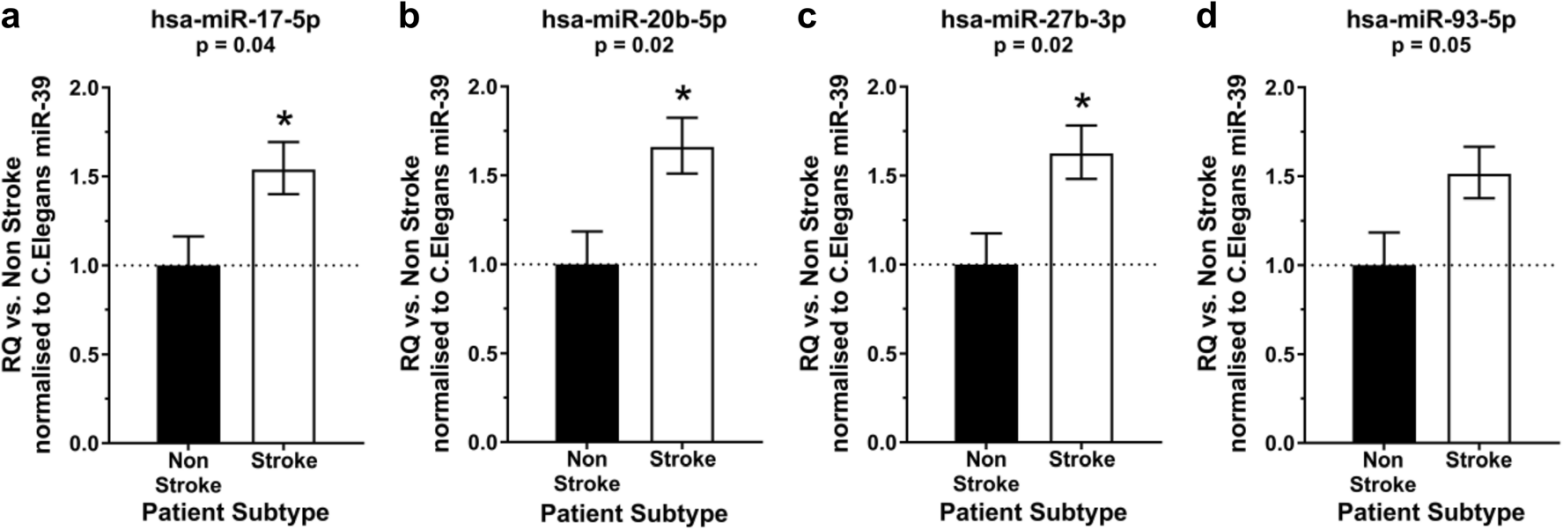
Expression of miRNAs in EV from stroke patients. The expression of hsa-miR-17-5p (**a**), hsa-miR-20b-5p (**b**), hsa-miR-27b-3p (**c**) and hsa-miR-93-5p (**d**) was profiled in EV isolated from stroke patients (*n* = 139) and compared to expression in non-stroke patients (*n* = 34). Change in miRNA expression was assessed at 48 h post-stroke by qRT-PCR and RQ calculated from ΔΔCt following normalisation to a spike housekeeper miRNA, *cel-miR-39*, and compared to the control, non-stroke patients. Data are presented as RQ ± RQmax/RQmin. Probability values were calculated using unpaired Student’s *t* test vs. non-stroke control patients, *p < 0.05.

Of the 13 miRNAs taken forward, expression of miRNA-17-5p (RQ 1.54 ± 0.15 vs. 1.00 ± 0.16 (*t*_170_ = 2.1; *p* = 0.039); Fig. 3a), miRNA-20b-5p (RQ 1.66 ± 0.17 vs. 1.00 ± 0.19 (*t*_171_ = 2.4; *p* = 0.017); Fig. 3b) and miRNA-27b-3p (RQ 1.62 ± 0.16 vs. 1.00 ± 0.18 (*t*_169_ = 2.4; *p* = 0.018); Fig. 3c) were significantly increased in EV isolated from people with ischemic stroke compared to non-stroke controls with miRNA-93-5p approaching significance (RQ 1.51 ± 0.15 vs. 1.00 ± 0.18 (*t*_171_ = 2.0; *p* = 0.051); Fig. 3d). Within these validated miRNAs, 3 are within the miRNA-17 family (miRNA-17-5p, -20b-5p and -93-5p). The expression of the remaining nine miRNAs did not differ (Online Resource 2).

**Fig. 4:**
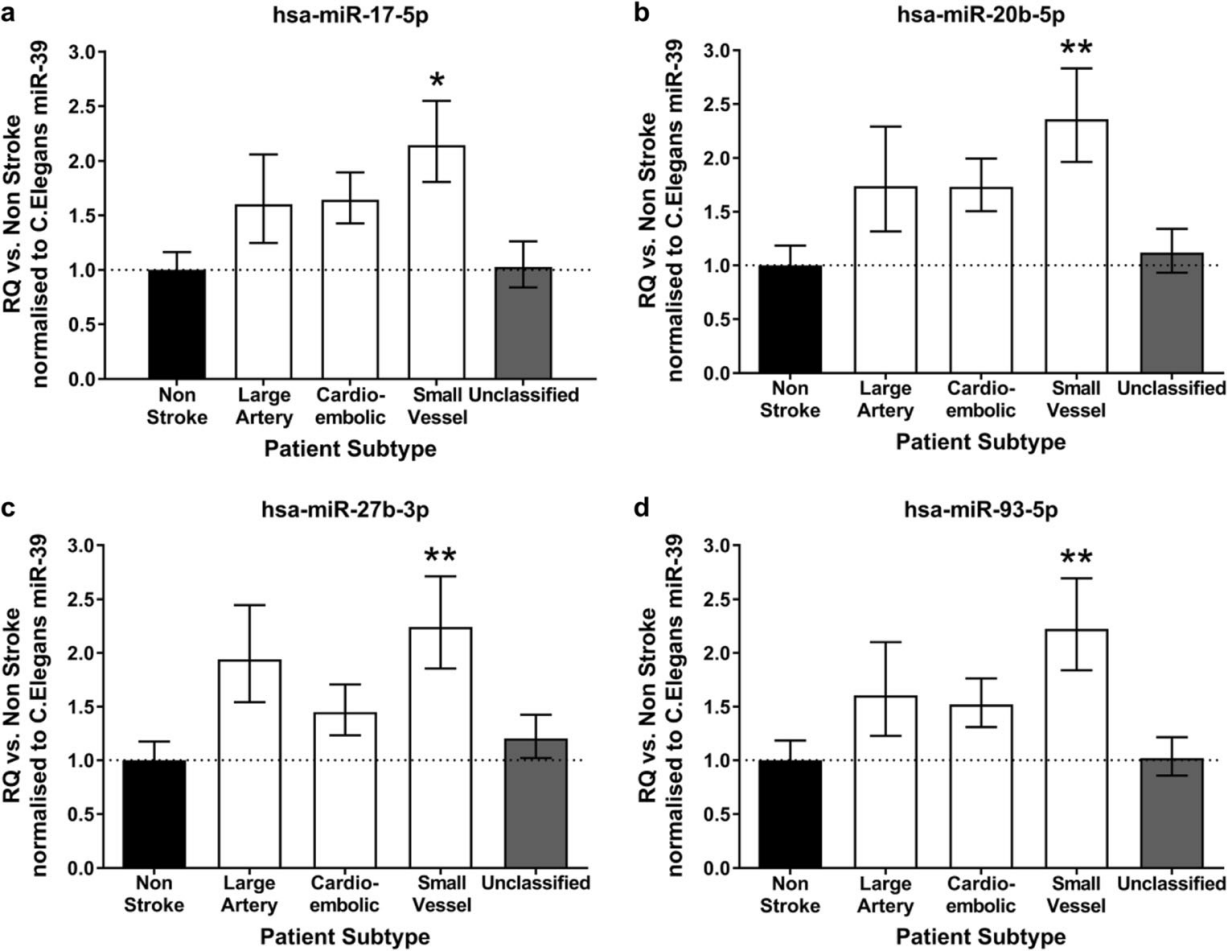
EV miR-17 family expression altered by stroke subtype. EV expression of hsa-miR-17-5p (**a**), hsa-miR-20b-5p (**b**), hsa-miR-27b-3p (**c**) and hsa-miR-93-5p (**d**) was profiled in all stroke patients (*n* = 139) of which there were large artery (*n* = 22), cardioembolic (*n* = 40), SVD (*n* = 37) and unclassified (*n* = 40). Expression was compared to that of control, non-stroke patients (*n* = 34). Change in miRNA expression was assessed at 48 h post-stroke by qRT-PCR and RQ calculated from ΔΔCt following normalisation to a spike housekeeper miRNA, *cel-miR-39* and compared to non-stroke control patients. Data are presented as RQ ± RQmax/RQmin. Probability values were calculated using one-way-ANOVA with post hoc Dunnett’s test, *p < 0.05; **p < 0.01

Expression of these four miRNAs (Fig. 3) differed by stroke subtype (Fig. 4). Expression was increased in patients with SVD stroke compared to non-stroke controls: miRNA-17-5p (RQ 2.15 ± 0.40 vs. 1.00 ± 0.16 (*F*_4,167_ = 3.5; *p* = 0.009)) (Fig. 4a), miRNA-20b-5p (RQ 2.36 ± 0.47 vs. 1.00 ± 0.19 (*F*_4,168_ = 3.9; *p* = 0.005)) (Fig. 4b), miRNA-27b-3p (RQ 2.24 ± 0.39 vs. 1.00 ± 0.15 (*F*_4,166_ = 3.4; *p* = 0.011)) (Fig. 4c) and miRNA-93-5p (RQ 2.23 ± 0.47 vs. 1.00 ± .18 (*F*_4,168_ = 3.5; *p* = 0.009)) (Fig. 4d). There were no differences between expression in other stroke subtypes and controls.

**Fig. 5:**
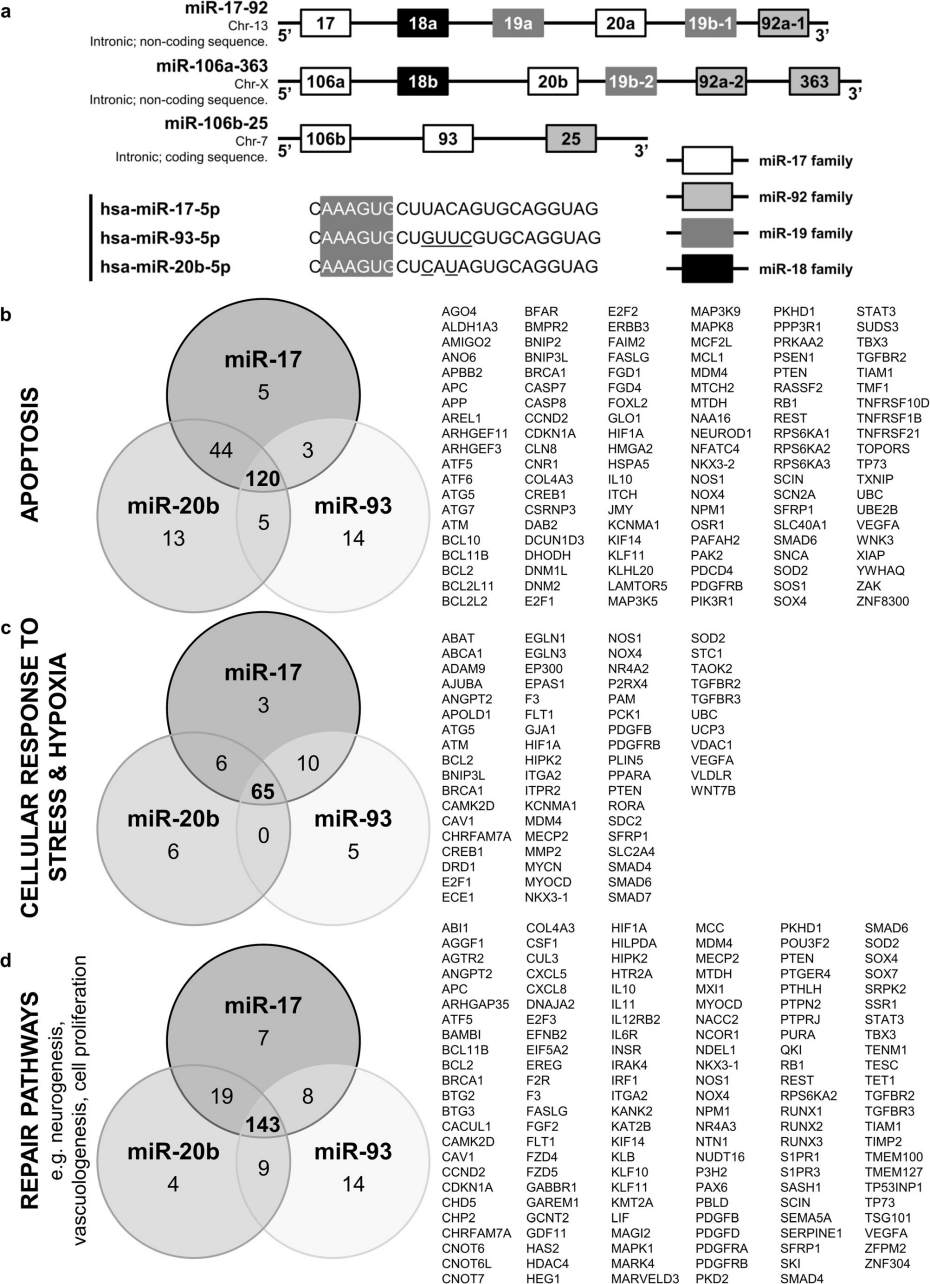
MiR-17 family miRNAs and targets. **a** Schematic representing three polycistronic miRNA clusters: miR-17-92, miR-106a-363 and miR-106b-25. The miRNAs in each cluster have been colour coded to represent which miRNA family (grouped according to seed sequence) each miRNA belongs to. The full miRNA structures for miRNAs -17-5p, -93-5p and -20b-5p have been given and the seed sequence (nucleotides 2–7) for each highlighted. miRNA targets were primarily selected on the basis of their complementarity to the seed sequence of any miRNA. The number of predicted targets involved in apoptotic pathways (**b**), cellular response to stress and hypoxia pathways (**c**) and repair pathways, including neurogenesis and vasculogenesis (**d**) have been summarised for each miRNA in the miR-17 family in Venn diagram form. The list of gene targets summarises those targets that all 3 miRNAs have in common

Bioinformatic analysis of predicted target genes for the miR-17 family (Fig. 5a) demonstrated many were involved in pathways related to stroke damage, for example apoptotic pathways (Fig. 5b), the response to stress/hypoxia (Fig. 5c) and repair pathways, including neurogenesis and vasculogenesis (Fig. 5d).

### Selected miRNA-17 Family miRNAs Altered in SHRSP vs. WKY

**Fig. 6:**
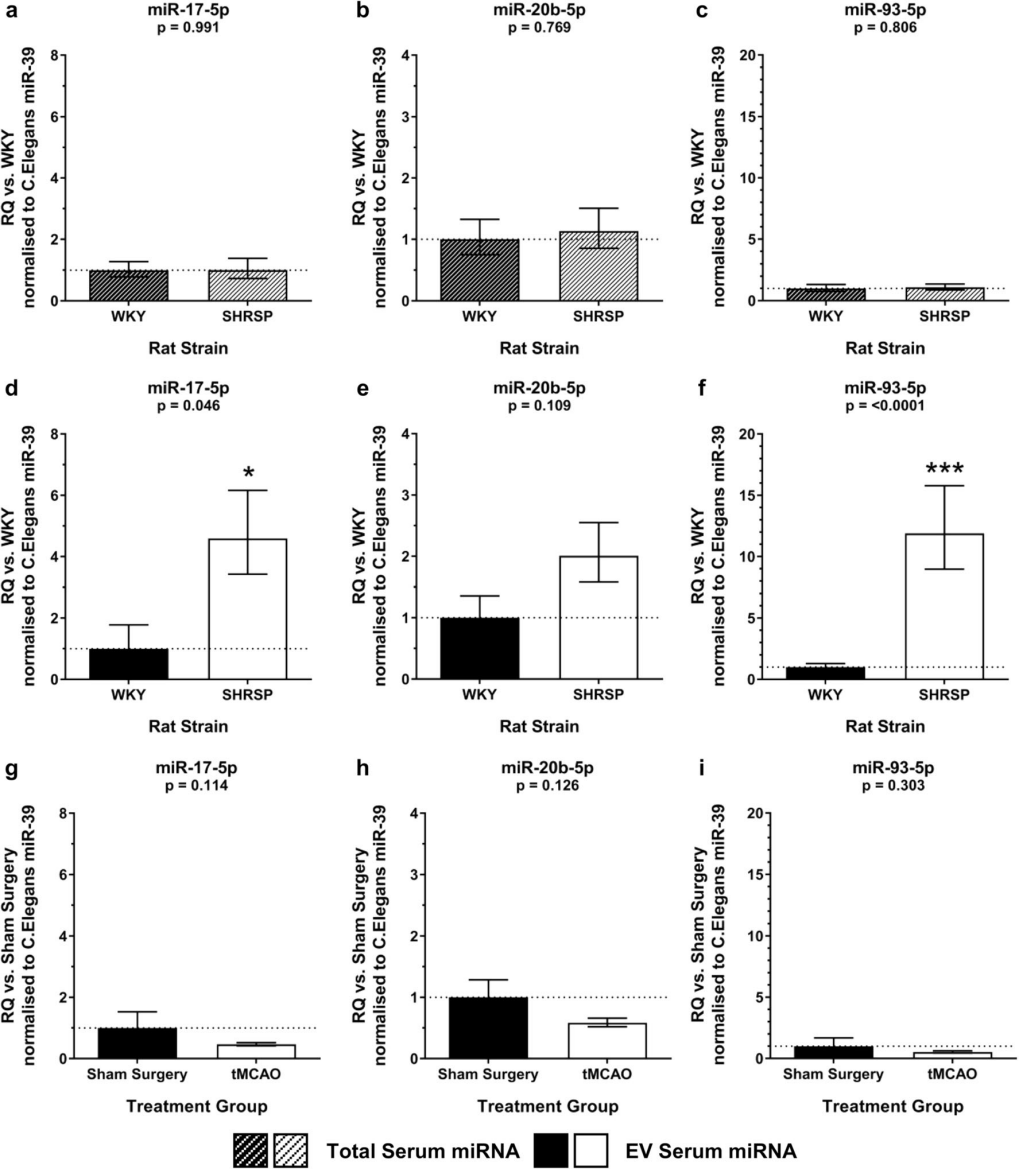
Circulating miR-17 family expression in naïve and post-tMCAO rats. Total serum expression of miR-17-5p (**a**), miR-20b-5p (**b**) and miR-93-5p (**c**) was assessed in naïve normotensive WKY and hypertensive SHRSP rats (*n* = 5–7/group). EV miRNA expression of miR-17-5p (**d**), miR-20b-5p (**e**) and miR-93-5p (**f**) was assessed in naïve WKY and SHRSP rats (*n* = 5–7/group). EV expression of miR-17-5p (**g**), miR-20b-5p (**h**) and miR-93-5p (**i**) was assessed in serum of SHRSP rats at 24 h following tMCAO (*n* = 3) or sham surgery (*n* = 3). Change in miRNA expression was assessed by qRT-PCR and RQ calculated from ΔΔCt following normalisation to a spike housekeeper miRNA, *cel-miR-39*, and compared to control (WKY or sham surgery) rats. Data are presented as RQ ± RQmax/RQmin. Statistical probability of differences in expression observed were calculated using unpaired two-tailed Student’s *t* test vs. control rats: *p < 0.05; ***p < 0.001

Characterisation of the circulating EV (Online Resource 3) in naïve WKY and SHRSP demonstrated no significant difference in the concentration or size of EV in naïve WKY vs. SHRSP (Online Resource 3b, c). miRNA-17-5p, -20b-5p and -93-5p, belonging to the miRNA-17 family (Fig. 5a), were the focus of the preclinical experiments. Total serum expression of the three miRNA-17 family miRNAs did not differ between naïve WKY and SHRSP rats (Fig. 6a–c) but EV expression of miRNA-17-5p (RQ 4.59 ± 1.57 vs. 1.00 ± 0.78 (*t*_8_ = 2.4; *p* = 0.046) (Fig. 6d)) and miRNA-93-5p (RQ 11.89 ± 3.88 vs. 1.00 ± 0.29 (*t*_12_ = 6.5; *p* < 0.0001)) (Fig. 6f)) was significantly increased in EV from naïve SHRSP vs. WKY. There was no significant difference in EV expression of miRNA-20b-5p between naïve SHRSP and WKY (RQ 2.01 ± 0.54 vs. 1.00 ± 0.36 (*t*_8_ = 1.8; *p* = 0.109) (Fig. 6e)).

### miRNA-17 Family miRNAs Are Not Altered in SHRSP EV Post-tMCAO

Experimental stroke in SHRSP resulted in a significant increase in the concentration, but not size, of circulating EV, 24 h after tMCAO compared to naïve WKY rats but not naïve SHRSP (Online Resource 3b, c). miRNA-17 family EV miRNA was not increased following tMCAO in comparison to sham operation in SHRSP rats: miRNA-17-5p (RQ 0.47 ± 0.05 vs. 1.00 ± 0.52, (*t*_4_ = 2.2; *p* = 0.11)) (Fig. 6g), miRNA-20b-5p (RQ 0.58 ± 0.08 vs. 1.00 ± 0.29, (*t*_4_ = 1.9; *p* = 0.13)) (Fig. 6h) and miRNA-93-5p (RQ 0.53 ± 0.10 vs. 1.00 ± 0.68, (*t*_4_ = 1.2; *p* = 0.30)) (Fig. 6i).

**Fig. 7:**
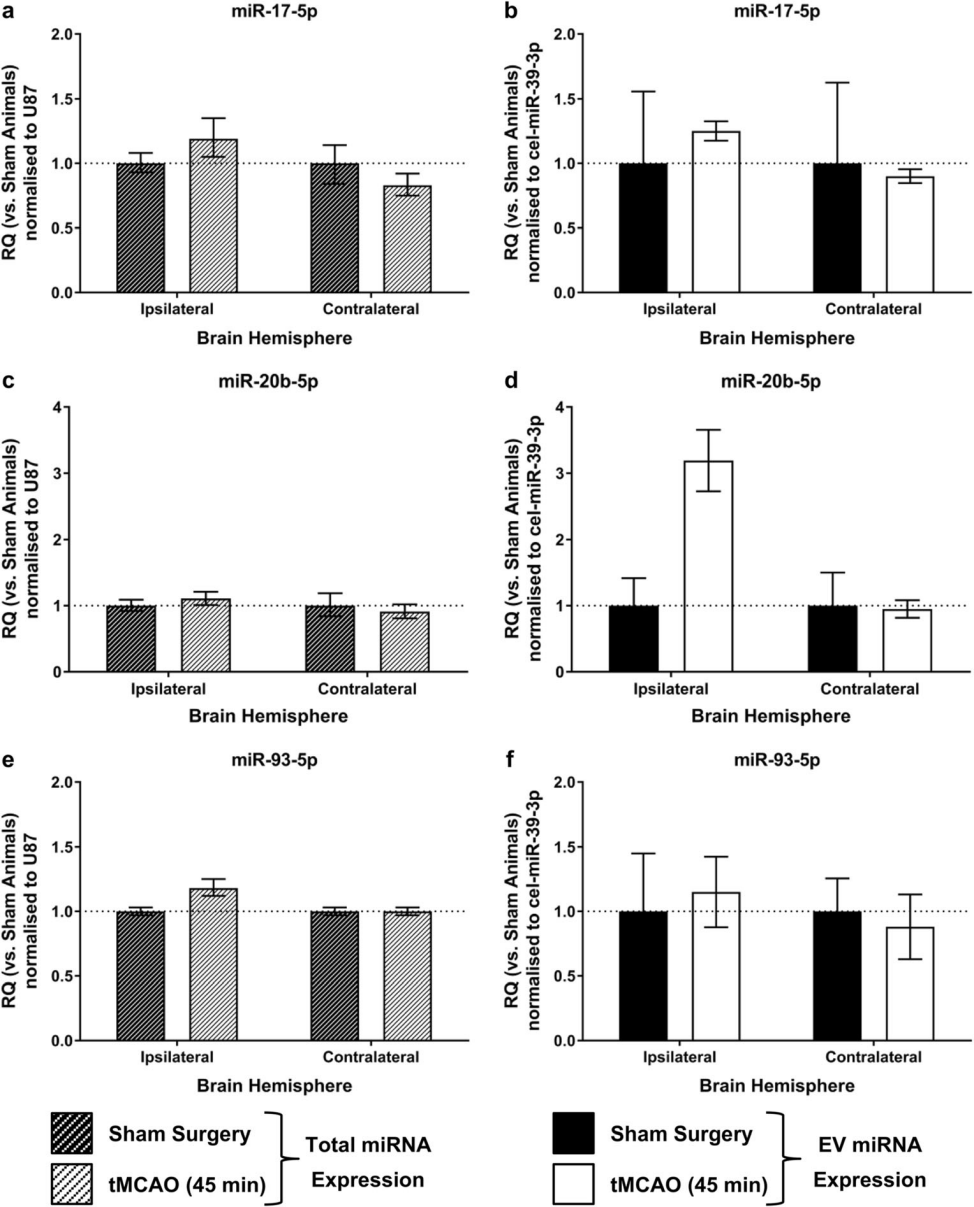
MiR-17 family expression in SHRSP brain or brain-derived EV post-tMCAO. miRNA expression was determined **a**, **c**, **e** peri-infarct tissue of SHRSP rats 24 h post-tMCAO or sham surgery (*n* = 6/group) or **b**, **d**, **f** brain-derived EV from peri-infarct tissue of SHRSP rats 24 h post-tMCAO or sham surgery (*n* = 3/group) for miR-17-5p (**a**, **b**); miR-20b-5p (**c**, **d**) or miR-93-5p (**e**, **f**). Change in miRNA expression was assessed by qRT-PCR and RQ calculated from ΔΔCt following normalisation to a spike in housekeeper miRNA, *cel-miR-39*, and compared to sham operated SHRSP rats. Data are presented as RQ ± RQmax/RQmin. Statistical probability of differences in expression observed were calculated using unpaired two-tailed Student’s *t* test with Bonferroni’s multiple comparisons test vs. sham-operated rats

There was no change in miRNA-17 family expression in peri-infarct tissue of tMCAO SHRSP compared to sham SHRSP (Fig. 7). The expression of miRNA-17-5p, -20b-5p and -93-5p were all unchanged in peri-infarct tissue and equivalent tissue in the contralateral hemisphere 24 h post-tMCAO: miRNA-17-5p (ipsilateral RQ 1.19 ± 0.16 vs. 1.00 ± 0.08 (*t*_9_ = 1.1; *p* = 0.31) and contralateral RQ 0.83 ± 0.09 vs. 1.00 ± 0.14 (*t*_10_ = 1.0; *p* = 0.34)) (Fig. 7a), miRNA-20b-5p (ipsilateral RQ 1.11 ± 0.10 vs. 1.00 ± 0.09 (*t*_9_ = 0.85; *p* = 0.41) and contralateral RQ 0.91 ± 0.11 vs. 1.00 ± 0.19 (*t*_10_ = 0.48; *p* = 0.64)) (Fig. 7c) and miRNA-93-5p (ipsilateral RQ 1.18 ± 0.07 vs. 1.00 ± 0.03 (*t*_9_ = 2.4; *p* = 0.039) and contralateral RQ 1.00 ± 0.03 vs. 1.00 ± 0.03 (*t*_10_ = 0.07; *p* = 0.95)) (Fig. 7e).

Brain-derived EV from the peri-infarct region and equivalent region in the contralateral hemisphere were of a comparable size (sham 111.8 ± 10.0 nm vs. tMCAO 134.6 ± 5.4 nm) to those found circulating in serum (Online Resource 3) with no difference between those isolated from sham SHRSP compared to SHRSP 24 h after tMCAO (*t*_10_ = 2.0; *p* = 0.073). Expression of miR-17 family expression (miRNA-17-5p, -20b-5p and -93-5p) was unchanged in brain-derived EV from peri-infarct material or the equivalent region on the contralateral hemisphere 24 h post-tMCAO: miRNA-17-5p (ipsilateral RQ 1.25 ± 0.08 vs. 1.00 ± 0.56 (*t*_4_ = 0.5; *p* = 0.65) and contralateral RQ 0.90 ± 0.05 vs. 1.00 ± 0.63 (*t*_4_ = 0.2; *p* = 0.84)) (Fig. 7b), miRNA-20b-5p expression was low and variable (ipsilateral RQ 3.19 ± 0.46 vs. 1.00 ± 0.42 (*t*_4_ = 3.1; *p* = 0.04) and contralateral RQ 0.95 ± 0.13 vs. 1.00 ± 0.50 (*t*_4_ = 0.12; *p* = 0.91)) (Fig. 7d) and miRNA-93-5p (ipsilateral RQ 1.15 ± 0.27 vs. 1.00 ± 0.45 (*t*_4_ = 0.34; *p* = 0.75) and contralateral RQ 0.88 ± 0.25 vs. 1.00 ± 0.25 (*t*_4_ = 0.39; *p* = 0.72)) (Fig. 7f).

## Discussion

We found and validated significant alterations in EV expression of four miRNAs in people with confirmed stroke compared to stroke mimics (non-stroke controls). Three of these miRNAs are part of the same family (miRNA-17 family). Differences were predominantly driven by increases in people with SVD stroke. In our pre-clinical study, we found increased expression of miRNA-17 family members in circulating compartmentalised EV in SHRSP compared to WKY but there was no increase in expression following experimental stroke. These findings raise the possibility that the differences seen in miRNA-17 expression in our stroke patients reflect the underlying pathobiology of cerebrovascular and SVD rather than the acute ischemic stroke (AIS) itself.

There are several differences between our study and previous stroke studies of miRNA expression. First, we enrolled people with suspected ischemic stroke and then established whether they had suffered a stroke or were stroke mimics. Thus, in contrast to many other clinical studies, the non-stroke patient cohort were not ‘healthy’. This increases the clinical relevance of any findings and makes it more likely the findings reflect cerebrovascular disease rather than differences in baseline factors or co-morbid conditions. Groups were well balanced regarding baseline characteristics such as age, sex, several key stroke-related risk factors and prescribed medications. This is crucial given the effect of age and sex on miRNA expression [21, 22]. Furthermore, within our study, we found no association between miRNA-17 family expression levels and stroke risk factors (e.g. age, sex, diabetes). Previous reports of miRNA expression in EV of stroke patients are limited by sample size, selection of controls, differences in baseline characteristics and a targeted approach to miRNA selection [4, 7, 11, 12]. We performed an unbiased screen. Interestingly, the number of EV isolated from serum of stroke patients and non-stroke controls did not differ here, in contrast to other studies [11, 23]. This may reflect differences in baseline factors between groups or time to sample collection from symptom onset. In previous studies, sampling was at 16.5 h [11] or 24 h [23]; while in our study, sampling was at 48 h after symptom onset. Interestingly, in our preclinical animal model, there was no difference in the size or number of circulating EV in control (WKY) and hypertensive (SHRSP) animals. When experimental stroke was performed on the SHRSP rats, the number of EV was significantly increased compared to normotensive WKY but not naïve SHRSP. The size remained unchanged. This supports that in the SHRSP, a model of SVD, there are no differences in EV burden compared to the reference strain, similar to what we see clinically in our heterogenous patient population. The lack of increase after AIS clinically, may reflect the presence of SVD in the non-stroke controls resulting in the failure to see the additional increase in EV number we see in the preclinical model when compared to the reference normotensive strain.

Of the 13 miRNAs selected for validation by qRT-PCR, 3 were significantly increased in a large AIS patient population: miRNA-17-5p, -20b-5p and -27b-3p with -93-5p approaching significance (*p* = 0.051). Three of these miRNAs belong to the miRNA-17 family (miRNA-17-5p, -20b-5p and -93-5p). They are transcribed from different chromosomes (13, 7 and *X*, respectively) but have an identical seed sequence important for target recognition. Total miRNA-17 expression in serum (rather than EV-compartmentalised) has been shown to be altered in stroke patients compared to controls with either vascular risk factors or other neurological disorders [24]. However, there were differences in some important risk factors and sex in this study. Using microarray, total miRNA-17, -20b and -93 expression was increased in whole blood samples from people with stroke, particularly in those with SVD but these changes were not validated by qRT-PCR or other means, the sample size was low and controls were healthy [6]. We found that changes in EV-compartmentalised miRNA-17 family expression were greatest in people with SVD stroke in comparison to stroke mimics. We saw no significant difference in people with large artery, cardioembolic and unclassified stroke.

In our preclinical animal model, levels of miRNA-17 family miRNA (miRNA-17-5p and -93-5p) expression was increased in circulating EV from SHRSP compared to the normotensive WKY strain. However, miRNA-17-5p and -93-5p levels did not increase further as a result of experimental stroke in SHRSP in either circulating EV, in the peri-infarct region or in brain-derived EV isolated from the peri-infarct region following tMCAO. This suggests the changes in miRNA-17 family expression reflect the development of cerebrovascular disease, rather than the acute stroke itself or that the miRNAs have reached a ceiling level in EV in SHRSP. Alternatively, it may reflect the different time points samples were taken clinically vs. preclinically with clinical samples collected at 48 h after AIS whilst preclinical samples were collected at 24 h after tMCAO. It is recognised that no animal model definitively reflects human SVD [25] but the SHRSP recapitulates many features: it demonstrates modest endothelial and perivascular defects, overactive microglia, poor myelination, non-specific inflammation and blood brain barrier (BBB) changes [26, 27] while lacking white matter hyperintensities on MRI or vasculopathy in penetrating arterioles [28]. Taken together with our clinical findings, it is possible that increased miRNA-17 family expression reflects the development of SVD, rather than the stroke itself.

Bioinformatic analysis of target genes for the miRNA-17 family members using the Database for Annotation, Visualisation and Integrated Discovery (DAVID; https://david.ncifcrf.gov/) shows several validated gene targets that may be involved in either stroke pathophysiology or cerebrovascular disease. These included targets linked to programmed cell death and apoptosis, the cellular response to stress and repair processes such as proliferation, axonogenesis and angiogenesis. We did not assess changes in target gene expression in the study as we did not have access to appropriate tissues.

We also validated changes in circulating levels of EV-packaged miRNA-27b-3p in the large patient cohort. Again, changes were most pronounced in those with SVD stroke. Previously, total miRNA-27b-3p was detected, but not increased, in plasma and cerebrospinal fluid in patients with stroke (*n* = 10; 3 days post-event) compared to those with other neurological diseases (*n* = 10) [29] although not in the EV compartment.

Previous studies have demonstrated changes in packaged circulating miRNAs: miRNA-9, -124 [11], -125b, -422a [13], -223 [12] -21 and -30a [7] in people with stroke. Of these, only miRNA-30a-5p and -223-5p demonstrated altered (increased) expression in circulating EV in our OpenArray™ but failed validation by qRT-PCR (*p* = 0.06 and 0.057, unpaired *t* test and Mann-Whitney *U* test, respectively). Differences in the control group (not matched for all CV risk factors), the time/type of blood sampling and EV/exosome isolation method may account for the disparities between the results of these studies [7, 12] and the current study. In addition, Chen et al. [12] did not stipulate if they determined changes in miRNA-223-3p or -5p strand and so this may account for the differences in changes reported between the studies.

The strengths of our study include the unbiased approach to the OpenArray™, validation, control selection and back translation to preclinical studies. Our study has limitations: in the OpenArray™, the low sample input protocol had to be used due to low RNA yields from patient EV samples (10 ng vs. standard 100 ng input). Thermo Fisher report that using 10 ng of input RNA with their adapted protocol will result in detection of 92% of miRNAs detected using 100 ng input RNA [18]. Furthermore, the OpenArray™ Human Panels each contain 754 unique human miRNA sequences, only a portion of the total number of miRNAs known to exist (2588 human sequences listed on miRbase http://www.mirbase.org/cgi-bin/mirna_summary.pl?org=hsa). It is possible, therefore, that we have missed changes in potentially important miRNAs. Normalisation of microarray and qRT-PCR data remains challenging for circulating RNA expression as there is not a universal reference gene or miRNA for normalisation [30]. Use of an external control for the OpenArray™ (*A. thaliana miRNA-159a*) or qRT-PCR (*C. elegans miRNA-39*) corrected for differences in qPCR efficiency but does not take into account endogenous differences in miRNA expression. However, due to the lack of a suitable endogenous control, the external control used was appropriate.

The SHRSP is the best available spontaneous model of human SVD [27, 31, 32] but the model has limitations. SHRSP demonstrate modest endothelial and perivascular defects as early as 5 weeks of age including impaired endothelial tight junctions, overactive microglia and poor myelination. They also have non-specific inflammation and blood brain barrier (BBB) changes [32] which may not be apparent in sporadic human SVD. We cannot be sure that the differences in expression in the SHRSP vs. WKY rat reflect SVD as identified in our human samples.

In summary, we have identified and validated changes in circulating EV miRNA expression in people with stroke and in particular, in people with SVD. Increased expression of multiple miRNAs from the miRNA-17 family was demonstrated. Similar findings were seen in SHRSP compared to WKY but experimental stroke did not alter expression. We hypothesise that changes in miRNA expression reflect the development of cerebral SVD rather than an acute stroke. Further studies to confirm the role of the miRNA 17 family are needed.

## Electronic Supplementary Material

ESM 1 (PDF 706 kb)
