# Supplementary material for: Altered Extracellular Vesicle MicroRNA Expression in Ischemic Stroke and Small Vessel Disease
Source: Transl Stroke Res. 2019 Jan 7;10(5):495–508. doi: 10.1007/s12975-018-0682-3 (PMC6733813; doi:10.1007/s12975-018-0682-3)
Supplement: Supplementary file 1 — (PDF 706 kb) [file 12975_2018_682_MOESM1_ESM.pdf]

ONLINE RESOURCES

ALTERED EXTRACELLULAR VESICLE MICRORNA EXPRESSION IN ISCHEMIC STROKE AND SMALL VESSEL DISEASE

Translational Stroke Research

Josie C. van Kralingen[1], Aisling McFall[1], Emily N.J. Ord[1], Thomas F. Coyle[1], Maria Bissett[1], John D. McClure[1], Christopher McCabe[2], I. Mhairi Macrae[2], Jesse Dawson[1], Lorraine. M. Work[1*]

[1]Institute of Cardiovascular & Medical Sciences, College of Medical, Veterinary and Life Sciences, University of Glasgow, Glasgow, UK

[2]Institute of Neuroscience & Psychology, College of Medical, Veterinary and Life Sciences; University of Glasgow, Glasgow, UK

[*]Corresponding Author:

Lorraine. M. Work

Lorraine.Work@glasgow.ac.uk

Online Resource 1 – Patient Characteristics by TOAST Subtype (Validation Study). Demographical data for the full cohort of patients used in the validation study, shown by TOAST stroke subtype. For dichotomous variables a Fisher's Exact Test was used to assess differences. For continuous variables a Kruskal Wallis Test ✞ was used. Abbreviations include ACE (angiotensin-converting enzyme), ARB (angiotensin receptor blocker), CCB (calcium channel blocker), NIHSS (National Institutes of Health Stroke Scale) and rtPA (recombinant tissue plasminogen activator).

		Non-Stroke	Large Artery	Cardioembolic	Small Vessel	Unclassified	p (Fisher's Exact Test)
	N	34	22	40	37	40	n/a
**Age & Gender**	Male, % (n)	55.9 (19)	77.3 (17)	70.0 (28)	70.3 (26)	47.5 (19)	0.082
	Median Age [IQR]	63.5 [53.8 – 68.0]	62.5 [50.5 – 73.3]	75.0 [65.0 – 79.8]	64.0 [54.0 – 73.0]	67.0 [55.0 – 76.8]	0.0012✞
**Risk Factors for Ischaemic Stroke**	Hypertension, % (n)	38.2 (13)	36.4 (8)	45.0 (18)	32.4 (12)	47.5 (19)	0.568
	Atrial Fibrillation, % (n)	8.8 (3)	0.0 (0)	60.0 (24)	2.7 (1)	5.0 (2)	<0.0001
	Diabetes (Type 1 or 2), % (n)	20.6 (7)	13.6 (3)	20.0 (8)	13.5 (5)	20.0 (8)	0.886
	Hyperlipidaemia, % (n)	17.6 (6)	13.6 (3)	30.0 (12)	21.6 (8)	32.5 (13)	0.367
	Peripheral Vascular Disease, % (n)	5.9 (2)	0.0 (0)	0.0 (0)	0.0 (0)	2.5 (1)	0.322
	Smoker, % (n)	26.5 (9)	54.5 (12)	25.0 (10)	37.8 (14)	22.5 (9)	0.084
	Ex Smoker, % (n)	32.4 (11)	22.7 (5)	15.0 (6)	10.8 (4)	20.0 (8)	0.238
	Previous Stroke, % (n)	14.7 (5)	9.1 (2)	7.5 (3)	18.9 (7)	17.5 (7)	0.524
	Family History, % (n)	11.8 (4)	4.5 (1)	10.0 (4)	18.9 (7)	25.0 (10)	0.207
**Medication**	rtPA, % (n)	n/a	36.4 (8)	25.0 (10)	27.0 (10)	27.5 (11)	0.819
	ACE Inhibitor, % (n)	23.5 (8)	22.7 (5)	17.5 (7)	24.3 (9)	27.5 (11)	0.898
	Alpha Blocker, % (n)	0.0 (0)	4.5 (1)	0.0 (0)	0.0 (0)	0.0 (0)	0.129
	Anticoagulant, % (n)	5.9 (2)	4.5 (1)	15.0 (6)	2.7 (1)	0.0 (0)	0.047
	Antiplatelet, % (n)	41.2 (14)	31.8 (7)	47.5 (19)	24.3 (9)	40.0 (16)	0.272
	ARB, % (n)	2.9 (1)	9.1 (2)	7.5 (3)	2.7 (1)	7.5 (3)	0.710
	Beta Blocker, % (n)	29.4 (10)	22.7 (5)	42.5 (17)	16.2 (6)	17.5 (7)	0.054
	Blood Pressure Treatment, % (n)	41.2 (14)	45.5 (10)	55.0 (22)	37.8 (14)	55.0 (22)	0.475
	CCB, % (n)	14.7 (5)	18.2 (4)	20.0 (8)	13.5 (5)	20.0 (8)	0.935
	Loop Diuretic, % (n)	2.9 (1)	4.5 (1)	15.0 (6)	13.5 (5)	7.5 (3)	0.339
	Oral Hypoglycaemic Drugs, % (n)	5.9 (2)	4.5 (1)	7.5 (3)	2.7 (1)	12.5 (5)	0.614
	Spironolactone, % (n)	2.9 (1)	0.0 (0)	2.5 (1)	2.7 (1)	0.0 (0)	0.807
	Statin, % (n)	47.1 (16)	50.0 (11)	52.5 (21)	29.7 (11)	42.5 (17)	0.310
	Thiazide, % (n)	8.8 (3)	18.2 (4)	10.0 (4)	2.7 (1)	15.0 (6)	0.291
**Stroke Status**	Median Baseline NIHSS [IQR]	1 [0-3]	7 [2-12.25]	4.5 [2-8]	4 [2-6]	3 [1-5]	<0.0001✞

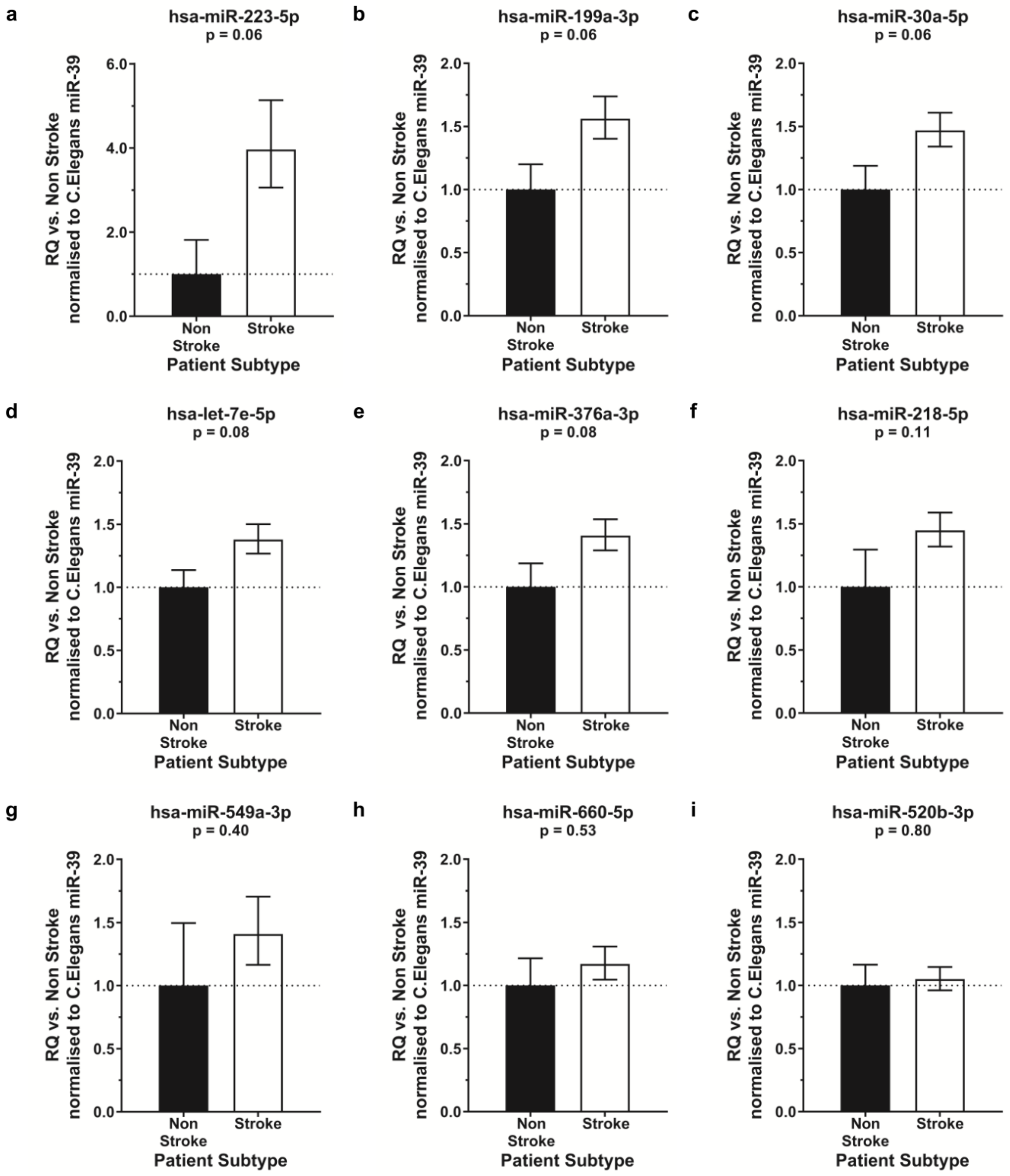

Online Resource 2 - Expression of miRNAs in EVfrom Stroke Patients. The expression of hsa-miR-223-5p **(a)**, hsa-miR-199a-3p **(b)**, hsa-miR-30a-5p **(c)**, hsa-let-7e-5p **(d)**, hsa-miR-376a-3p **(e)**, hsa-miR-218-5p **(f)**, hsa-miR-549a-3p **(g)**, hsa-miR-660-5p **(h)** and hsa-miR-520b-3p **(i)** was profiled in EV isolated from stroke patients (n=139) and compared to expression in non-stroke patients (n=34). Change in miRNA expression was assessed at 48 hours post-stroke by qRT-PCR and relative quantification (RQ) calculated from ΔΔCt following normalisation to a spike housekeeper miRNA, *cel-miR-39*, and compared to miRNA expression in the non-stroke control patients. Data shown are RQ ± RQmax/RQmin. Probability values were calculated using unpaired Student's t-test (or Mann-Whitney U test where appropriate), vs. non-stroke control patients.

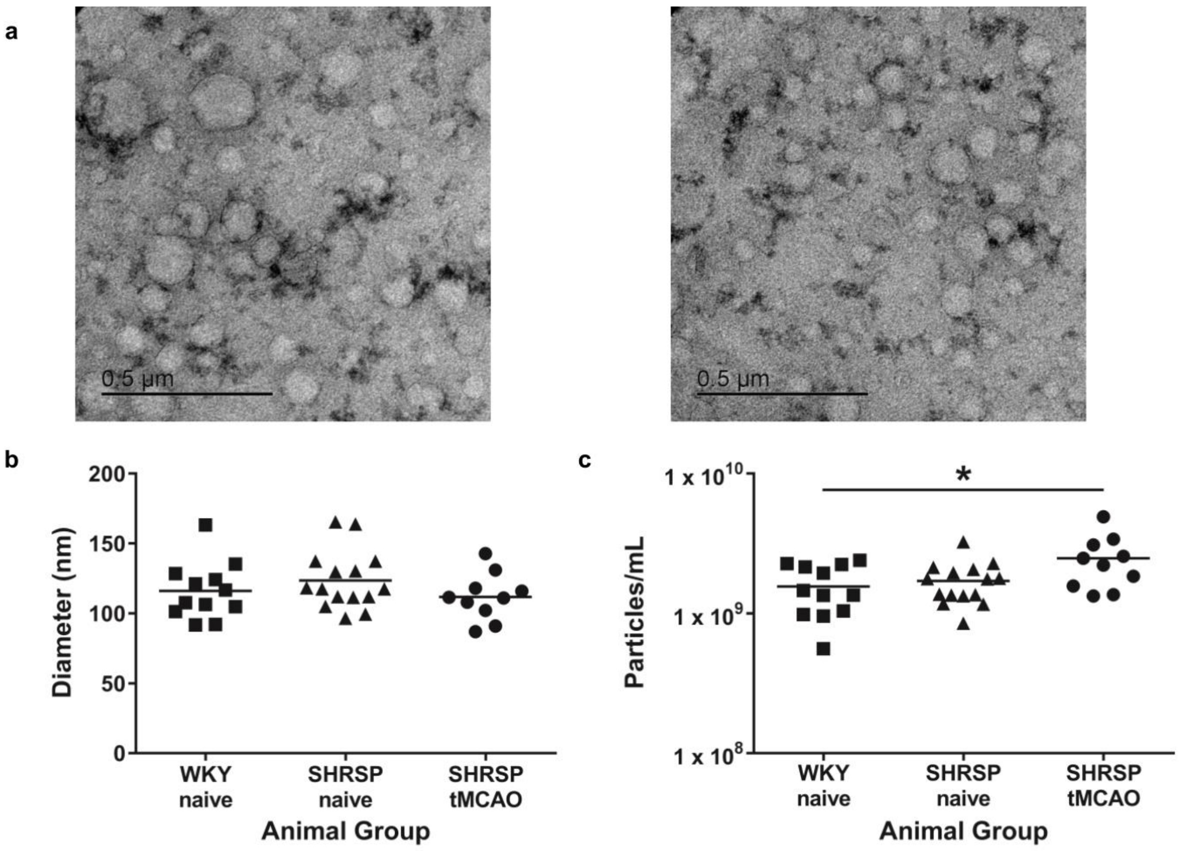

Online Resource 3 - Preclinical EV Visualisation and Quantification. **(a)** Electron-microscopic observation of whole-mounted EV purified from naïve SHRSP serum by precipitation. Representative images of EV isolates from naive SHRSP rat samples obtained by transmission electron microscopy showing both larger microvesicle and exosome sized particles. The average diameter (nm) of EV **(b)** and their concentration (measured by number of particles/mL) **(c)** was assessed in serum from naïve WKY (n=12) and SHRSP (n=15) as well as serum sampled from SHRSP rats at 24 hours following a 45 min tMCAO (n=10). The horizontal bar represents the mean. Statistical probability of differences in diameter or concentration observed were calculated using one-way-ANOVA with post-hoc Tukey's test, *p<0.05.
